# Cango Lyec (Healing the Elephant): Chronic Hepatitis B Virus among post-conflict affected populations living in mid-Northern Uganda

**DOI:** 10.1371/journal.pone.0251573

**Published:** 2021-05-27

**Authors:** Samuel S. Malamba, Herbert Muyinda, D. Martin Ogwang, Achilles Katamba, David S. Zamar, Kate Jongbloed, Nelson K. Sewankambo, Martin T. Schechter, Patricia M. Spittal

**Affiliations:** 1 HIV Reference Laboratory Program, Uganda Virus Research Institute (UVRI), Entebbe, Uganda; 2 Child Health Development Center, Makerere University, Kampala, Uganda; 3 St Mary’s Hospital–Lacor, Gulu, Uganda; 4 Northern Uganda Program on Health Sciences, Gulu, Uganda; 5 College of Health Sciences, Makerere University, Kampala, Uganda; 6 School of Population and Public Health, University of British Columbia, Vancouver, BC, Canada; 7 BC Children’s Hospital Research Institute, Vancouver, BC, Canada; University of Cincinnati College of Medicine, UNITED STATES

## Abstract

**Background:**

The legacy of war in Northern Uganda continues to impact people’s health and wellbeing in the Acholi region. Despite increasing attention to Hepatitis B Virus (HBV) in Uganda and globally, concerns remain that unique drivers of infection, and barriers to screening, and treatment, persist among those affected by conflict.

**Methods:**

Cango Lyec (Healing the Elephant) cohort survey involved conflict-affected adults aged 13–49 in three mid-Northern Uganda districts (Gulu, Amuru and Nwoya). Baseline (2011–2012) samples were tested for HBV surface antigen (HBsAg), HBV e-antigen (HBeAg), antibodies to HBV surface antigen (HBsAb), antibodies to HBV e-antigen (HBeAb), and antibodies to HBV core antigen (HBcAb). All HBsAg positive samples were tested for IgM antibodies to HBV B core antigen (HBc-IgM) and where available, >6-month follow-up samples were tested for HBeAg and HBV DNA. Data were analyzed using STATA 15 software. Logistic regression accounted for variance due to complex two-stage sampling that included stratification, unequal selection probabilities and community clustering. Odds ratios measured effect potential risk factors associated with chronic HBV infection.

**Results:**

Among 2,421 participants, 45.7% were still susceptible to HBV infection. HBsAg seropositivity was 11.9% (10.9–13.0), chronic HBV was 11.6% (10.4–12.8), acquired immunity resulting from vaccination was 10.9%, and prior natural infection was 31.5%. Older age (OR:0.570; 95%CI:0.368–0.883) and higher education (OR:0.598; 95%CI:0.412–0.868) were associated with reduced odds of chronic HBV infection. Being male (OR:1.639; 95%CI:1.007–2.669) and having been abducted (OR:1.461; 95%CI:1.055–2.023) were associated with increased odds of infection. Among women, having 1 or 2 pregnancies (compared to none or >2) was associated with increased odds of infection (OR:1.764; 95%CI:1.009–3.084).

**Conclusion:**

Chronic HBV is endemic in Gulu, Amuru and Nwoya districts. Recommended strategies to reduce post-conflict prevalence include establishment of Northern Uganda Liver Wellness Centres, integration of screening and treatment into antenatal care, and roll out of birth-dose vaccination.

## Introduction

### Hepatitis B: Global, sub-Saharan Africa, and Uganda

Chronic Hepatitis B Virus (HBV) infection is defined as persistence of Hepatitis B surface antigen (HBsAg) for six or more months after new onset of HBV infection. Despite being vaccine-preventable, it is estimated there are 240 million chronic HBV carriers, of whom 650,000 die from complications of the infection each year [1, 2]. Chronic HBV causes liver disease, and can accelerate its progression to cirrhosis and liver cancer, as well as modify HIV disease progression and response to treatment [3–10]. HBV-related mortality has been increasing steadily and, along with other viral hepatitis infections, is now considered one of the top global public health threats [9, 11]. In Sub-Saharan Africa, estimated overall prevalence of HBV surface antigen remains high at 6·1% [9]. Prevalence of HBV infection is slightly lower in Uganda, at an estimated 4.1%; 5.4% among men and 3.0% among women [12]. National Ugandan estimates also indicate that HBV infection is higher among people living with HIV, compared to those without HIV [4, 12]. The 2016 World Health Organization strategy on viral hepatitis has established a goal to reduce new cases of chronic HBV by 90% and mortality due to HBV by 65% by 2030 [11]. The Ugandan Ministry of Health declared HBV a public health priority in 2014 and made commitments to scale up testing and vaccination starting in 2015 [8]. Despite increasing attention to HBV infection within Uganda and globally, there is concern that unique drivers of infection, as well as barriers to screening and treatment among key populations, will persist if they are not adequately understood [13].

### Northern Uganda: HBV in the post-conflict context

For over two decades (1986–2006), people of Northern Uganda suffered a protracted war between the Government of Uganda and Lord’s Resistance Army. The war was characterized by violence, abductions, displacement, destruction of infrastructure, social services, and weakening of the social and economic fabric of society. Legacy of prolonged civil war in Northern Uganda continues to have significant impacts on health and wellbeing of people living in the region, including concerns related to HBV. Evidence suggests that districts in Northern Uganda have considerably higher levels of HBV infection than other parts of the country and we have continued to see varying prevalence estimates of HBV infection in Northern Uganda over time [4, 12, 14]. The 2005 Uganda HIV/AIDS Sero-Behavioural Survey reported an HBsAg prevalence of 20.7% among 15–59 year-olds across the mid-Northern region [4]. In 2013, a population-based survey in Gulu Municipality, Northern Uganda reported HBsAg prevalence of 17.6% [14]. The recent 2016–17 Uganda Population-based HIV Impact Assessment (UPHIA) survey estimated HBsAg prevalence to be 4.6% among participants aged 15–64 years in mid-Northern Uganda [12]. In the same survey, the observed prevalence among Acholi people was 9.2%, the highest among all ethnicities in the country [12]. While UPHIA involved rural and poorly served remote areas across Uganda, prevalence of HBV infection was highest in areas affected by the war [12].

Concerns are increasing that young adults (particularly young men) in the North are presenting to care with advance liver cancer and experiencing rapid progression to death. This is affirmed by recent evidence documenting liver cancer incidence of 12.8 (men) and 6.9 (women) per 100,000 population between 2013–2016 in the Northern region, which is higher than Kampala in Central Uganda as well as most sub-Saharan African countries [15]. The seemingly ‘sudden’ onset of symptoms and rapid decline in one’s health may be contributing to emergence of stigmas similar to those related to infectious diseases such as ebola and HIV [16]. These concerns are exacerbated by enduring impacts of the war on health infrastructure and access to health care services [17].

### Rationale & objectives

Whereas concerns remain that a high prevalence of HBV poses a significant burden in post-conflict Northern Uganda resulting from years of life lost to liver disease, the existing evidence does not differentiate between acute and chronic HBV [14]. We set out to test stored samples from the Cango Lyec (Healing the Elephant) study [18] to determine the proportion of study participants with chronic, acute, immune-natural or vaccine-related Hepatitis B Virus infection; the proportion of those who were still susceptible to HBV; and the chronic HBV-related vulnerabilities among post-conflict affected populations in Gulu, Nwoya and Amuru Districts, mid-Northern Uganda in order to inform future interventions.

## Methods

### Study design and sample

This analysis involved secondary data from a population based cross-sectional survey involving adults 13–49 years old. Study methods have previously been reported in detail [18]. Briefly, during the Cango Lyec survey a two-stage stratified sampling method was used to randomly select three study communities in each district, one from each settlement category. All communities in the three districts were listed; all major settlements were mapped and categorized as either ‘Permanent’ (settlements which were there before the war and residents were never displaced), ‘Transient’ (settlements created to accommodate internally displaced people (IDP) returning to their long-abandoned lands and destroyed houses) or ‘Displaced’ (settlements created to accommodate IDP during the war). All households in the selected communities were mapped and a household census was completed to establish the number and demographic characteristics of all household members. A “take all” approach was used to survey all consenting individuals aged 13–49 years who had been residents in the selected communities for the last month. These baseline field activities were conducted between November 2011 and July 2012.

### Data collection and measures

Data were collected on sociodemographics, war-related experiences, sexual health and histories, and mental health, using interviewer-administered surveys translated from English into Luo through a process of forward-backward translation by an experienced team of health professionals working independently. Where there was disparity, translations were discussed and revised to achieve intended meaning. Venous blood was taken for hepatitis B virus, HIV and syphilis serology. This analysis included only those samples provided by participants who provided informed consent for interviews and blood tests at the time of the study and for future additional blood tests.

#### Laboratory testing

Hepatitis B serologic testing involved measurement of several HBV-specific antigens and antibodies [19]. All samples were tested for HBsAg, Hepatitis B e-antigen (HBeAg), HBsAb, antibodies to Hepatitis B e-antigen (HBeAb), and antibodies to Hepatitis B core antigen (HBcAb). The HBsAg was tested on two different kits for quality assurance and in all samples tested, there was 100% concordance.

Different serologic “markers” or combinations of markers were used to determine whether a patient had acute or chronic HBV infection, was immune to HBV as a result of prior infection or vaccination, or was susceptible to infection.

Testing was done according to the Uganda Ministry of Health Central Public Health Laboratory (CPHL) standard operating procedures and results were interpreted according to the US Center for Disease Control and Prevention (CDC) algorithm. After consultation with subject matter experts at Mulago National Referral Hospital, Uganda Virus Research Institute (UVRI) and CPHL, sample HBV results classified as unclear or difficult to interpret/classify were reclassified as coming from individuals who acquired immunity due to natural infection (Table 1).

**Table 1:** Interpretation of Hepatitis B Virus serology testing results.

	Baseline		Adjusted using Follow-up results	
**Total tested for HBV included in the analysis**	2,421	100.0	2,421	100.0
**Not exposed**	1,103	45.6	1,106	45.7
*(HbsAg-*, *Anti-HbcAb-)*				
**Acquired immunity due to natural infection**	245	10.1	251	10.4
*(HbsAg-*, *Anti-HbcAb+*, *Anti-HbsAb+)*				
**Acquired immunity due to vaccination**	264	10.9	264	10.9
*(HbsAg-*, *Anti-HbcAb-*, *Anti-HbsAb+)*				
**Acutely infected- infection less than 6 months old**	3	0.1	0	0.0
*(HbsAg+*, *Anti-HbcAb+*, *Anti IgM-HbcAb+*, *Anti-HbsAb-)*				
**Chronically infected**	268	11.1	278	11.5
*(HbsAg+*, *Anti-HbcAb+*, *Anti IgM-HbcAb-*, *Anti-HbsAb-)*				
Difficult to interpret, either resolved infection, acutely infected or low level of chronic infection *(HbsAg-*, *Anti-HbcAb+*, *Anti-HbsAb-)*	511	21.1	511	21.1
**Unclarified, most likely chronic infection**	12	0.5	1	0.0
**Unclassified**	15	0.6	10	0.4

HBsAg testing was performed using Abbott ARCHITECT HBsAg quantitative reagent kit, Architect HBsAg quantitative calibrator and Architect HBsAg quantitative controls. Total HBsAg were tested using the Vitros Chemiluminescence Immuno-assays (Ortho Clinical Diagnostics, Rochester, NY). The HBeAg testing to detect replication of HBV was applied to both HBsAg positive and negative specimens. This test was performed using ARCHITECT i2000SR, HBeAg Calibrator, control packs, pre-trigger solution and probe conditioning solution for the assays.

All HBsAg positive samples were tested for IgM antibodies to Hepatitis B core antigen (HBc-IgM) to identify acute infection and, viral load using the m2000sp/rt Abbott System to determine active viral load. HBcAb-IgM to detect recent HBV infection (within 6 months) was applied to HBsAg positive samples only. This test was also performed using ARCHITECT i2000SR, HBcAb-IgM Calibrator, control packs, pre-trigger solution and probe conditioning solution for the assays.

#### Primary outcome

The primary outcome of interest was chronic HBV infection, defined as having a positive surface antigen test (HBsAg) and HBcAb, with negative Anti IgM-HbcAb and Anti-HbsAb tests, or a sample with HBsAg+ with a >6 months follow-up sample having a positive surface antigen test (HBsAg+).

#### Study variables

Participant characteristics included age group (13–34 years/35-49 years), district of residence (Amuru/Gulu/Nwoya), marital status (not in union/currently in union), educational level (no education/ever been to school), and community displacement status (permanent/transient/displaced).

Three measures assessed exposure to war-related trauma and mental health sequelae. They were participants’ reports whether they had ever been abducted (yes/no), Luo versions of the Harvard Trauma Questionnaire (HTQ), and Hopkins Symptom Check List-25 (HSCL-25) assessment of mental health concerns. Cango Lyec’s use of these scales to measure experiences of traumatic events, probable post-traumatic stress disorder (PTSD) and probable depression has been described elsewhere in detail [18, 20, 21]. Immediate referrals to mental healthcare were made for participants whose scores met thresholds of concern on the HSCL-25 or HTQ, as well as those who reported feeling hopeless about the future, thoughts about ending their life, and feelings of worthlessness.

Women of child-bearing age (13–45) were asked about sexual history (never had sex/ever had sex), pregnancy history (never been pregnant/≤2 pregnancies/>2 pregnancies) and current pregnancy (yes /no).

### Analysis

Data were analyzed using STATA 15 software (StataCorp LP, 4905 Lakeway Drive, College Station, TX, USA). Logistic regression survey analysis procedures in STATA were used to account for the additional variance due to the complex two-stage sampling study design that included stratification by the three settlement types and three study districts, as well as the unequal selection probabilities and clustering. Odds ratios were used to measure the magnitude of the effect of factors associated with Chronic HBV infection in both men and women as well as vaccination among women of childbearing age. Sampling weights were used to adjust for design effects, ensuring that the estimates were representative of the study population. The sampling weights were based on the selection probabilities at each level of selection and on the proportion of survey participants consenting for interview and future testing of blood samples. For independent categorical variables, we included a missing value category to minimize list-wise deletion of observation in the models. Baseline sample characteristics between those who tested positive and those who tested negative for chronic HBV were compared using weighted, Rao-Scott adjusted Pearson’s chi-square test statistics. As this was an explorative study, and the variables of interest were specified a priori and likely to correlate with one another, correction for multiple testing was not performed [22].

### Ethical considerations and official approvals

Ethical approvals for the Cango Lyec cohort study were obtained from the University of British Columbia-Providence Healthcare Research Ethics Board (Canada), Makerere University College of Health Sciences-School of Public Health Ethics Committee (Uganda) and Uganda Virus Research Institute-Science and Ethics Committee (Uganda). The Uganda National Council for Science and Technology (Uganda) issued a letter of approval based on non-objection from the Office of the President of Uganda. Subsequently, the Resident District Commissioner in each district provided administrative approval affirming that the study is welcome to be carried out in that district. Informed written consent was also obtained from all eligible study participants aged 13–49 years after explaining the objectives and procedures of the study. Parental/legal guardian’s written consent for <18 years with minor’s assent were also obtained. Every participant was properly counselled and consented before being asked to sign or put a fingerprint on the consent form if they were not able to write. Study participants were assured of confidentiality before the start of each interview. All data collected was kept under lock and key only accessible to the research team.

## Results

**Fig 1:**
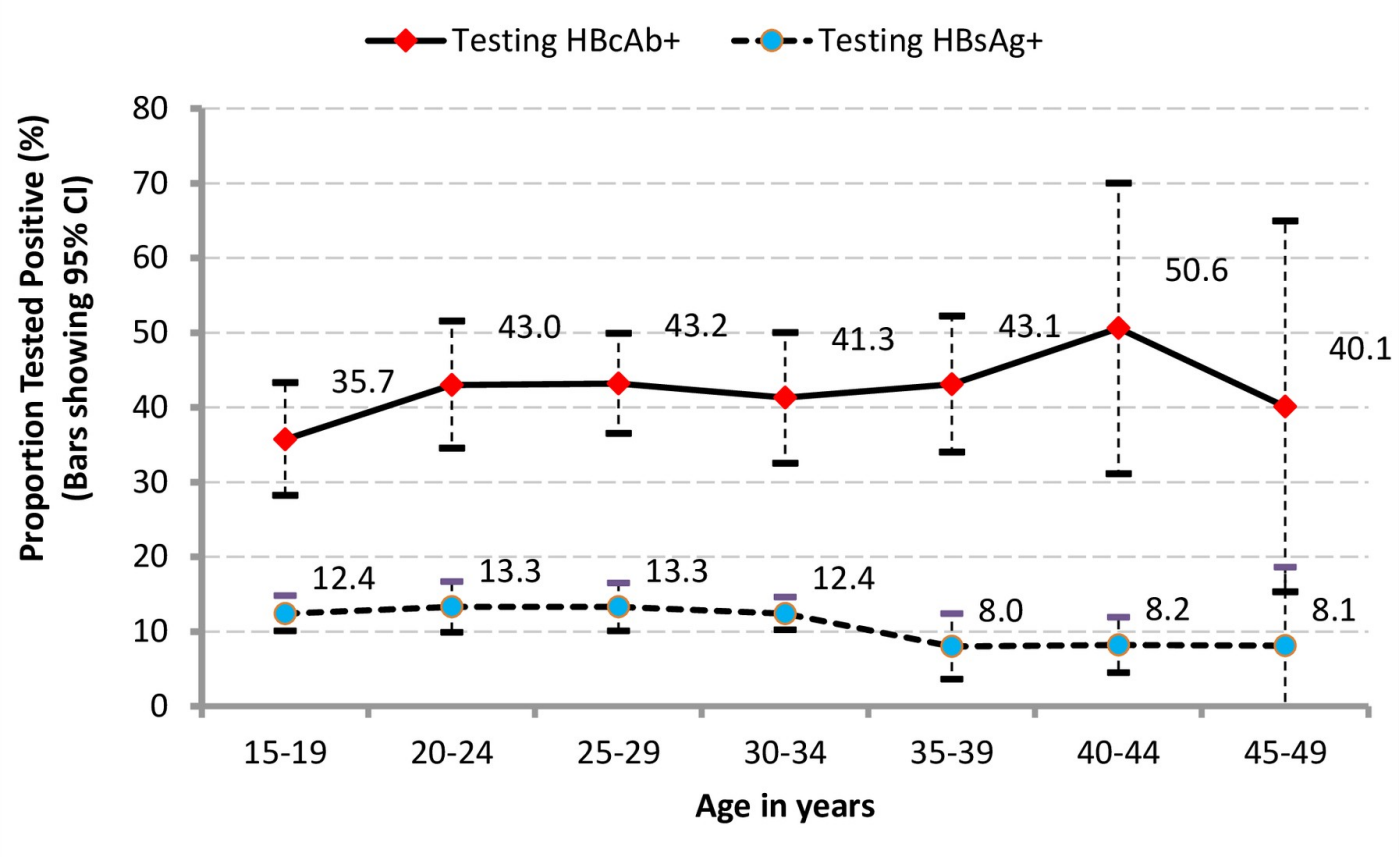
Sample flow diagram. Study participants’ sample testing flow diagram in the *Cango Lyec* study, mid-northern Uganda districts of Amuru, Gulu and Nwoya, 2011/2012. Approximately 81% of all eligible Cango Lyec participants and 99% of those who consented for future blood tests were analysed.

A total of 2,607 samples were collected during the baseline survey, of which 2,421 (93%) came from individuals who consented to testing of their blood sample to answer future research questions and were included in this analysis (Fig 1).

### Participant characteristics and prevalence of Hepatitis B exposure and infection

**Fig 2:**
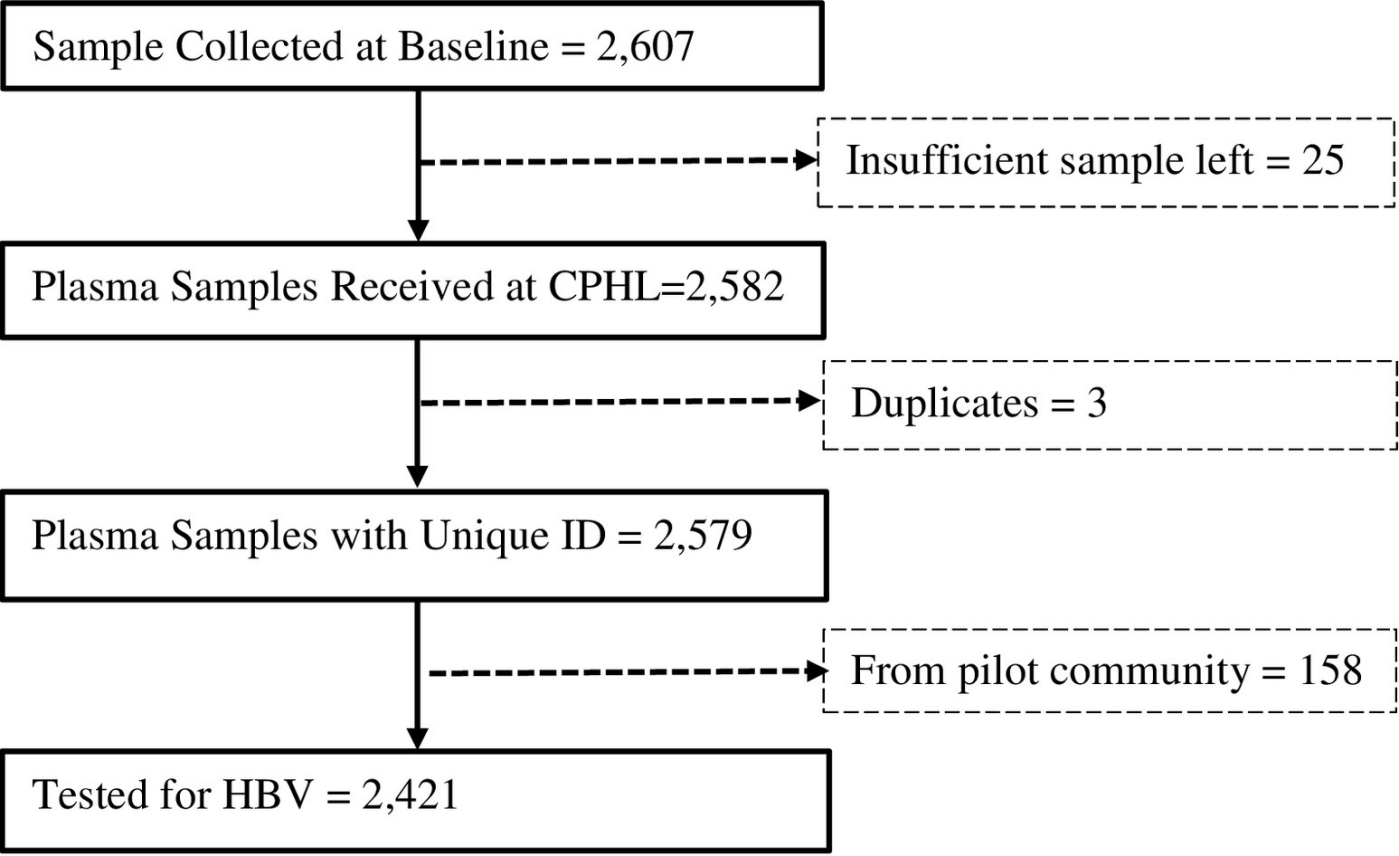
Proportion testing positive to HBcAb and to HBsAg by age, mid-northern Uganda districts of Amuru, Gulu and Nwoya 2011/12. †Lifetime exposure (♦HBcAb+) or Current acute or chronic Hepatitis B infection (●HBsAg+).

Prevalence of chronic HBV infection was 11.5% (n = 278) (Table 1). Just 3 infections were considered acute (acquired within the past six months) and prevalence of HBV vaccination was 10.9% (n = 264). The proportion who were HBV surface antigen positive (HBsAg+) remained relatively constant ranging between 12.4–13.3% across age-groups, until the 35–49 year age-group where it dropped to 8% (an almost 40% drop) (Fig 2). By the age of 15–19 years, 35.7% (95% CI: 28.2–43.3) of the population had been exposed to HBV (HBcAb+), a marker of lifetime exposure to HBV. Median viral load was 2.99 (IQR: 2.01–4.87), slightly lower among women (2.65; IQR: 1.30–3.53) compared to men (3.52; IQR: 2.43–6.81) (data not shown).

Among participants with chronic HBV infection, 145 were men (13.6%, 95%CI: 11.4–16.2) and 133 were women (10.3%, 95%CI: 8.6–12.4) (Table 2). Prevalence of chronic HBV infection was higher among young people 13–34 years old (12.5%, 95%CI: 10.9–14.3) compared to those aged 35–49 years (8.1%, 95%CI: 5.6–11.5), those not in marital unions (13.3%, 95%CI: 11.2–15.7) compared to those who were currently in a marital union (10.3%, 95%CI: 8.5–12.4), those with no formal education (15.0%, 95%CI: 11.2–29.7) compared to those who had ever been to school (11.2%, 95%CI: 9.7–12.8), and among women reporting a history of 1–2 pregnancies (14.7%, 95%CI: 10.9–19.5) compared to women who have never been pregnant (8.7%, 95%CI: 5.7–12.8) (Table 2).

**Table 2:** Chronic HBV infection by socio-demographic and medical history characteristics at baseline.

*Characteristics of participant*	*Total*	*Chronic HBV*	*Weighted*		
	N	n	% Positive	95% CI	P value
***Overall***	2,421	278	11.7	[10.3, 13.0]	
***Age group***					
*13–34 years*	1,945	242	12.5	[10.9, 14.3]	
*35–49 years*	476	36	8.1	[5.6, 11.5]	0.023
***Sex***					
*Female*	1,409	133	10.3	[8.6, 12.4]	
*Male*	1,012	145	13.6	[11.4, 16.2]	0.032
***Marital Status***					
*Not in union*	1,141	150	13.3	[11.2, 15.7]	
*Currently in union*	1,280	128	10.3	[8.5, 12.4]	0.048
***Education level***					
*No Education*	354	52	15.0	[11.2, 19.7]	
*Now or ever been to school*	2,067	226	11.2	[9.7, 12.8]	0.069
***District of residence***					
*Amuru*	709	70	10.4	[8.1, 13.2]	
*Gulu*	1,159	131	11.8	[9.8, 14.0]	
*Nwoya*	553	77	14.3	[11.6, 17.7]	0.203
***Community type***					
*Displaced*	329	32	10.1	[7.1, 14.3]	
*Permanent*	1,143	143	12.4	[10.5, 14.6]	
*Transient*	949	103	11.0	[8.7, 13.8]	0.495
***Ever been Abducted***					
*No*	1,833	199	11.0	[9.5, 12.8]	
*Yes*	588	79	14.1	[11.0, 17.7]	0.089
***Sexually abused in abduction***					
*No*	457	61	14.2	[10.8, 18.5]	0.410
*Yes*	99	10	10.6	[5.3, 20.0]	
***Sexual assault in context of war***					
*No*	2,216	262	11.9	[10.5, 13.6]	0.221
*Yes*	205	16	8.6	[5.0, 14.3]	
***Pregnancies***					
*Never been Pregnant*	332	29	8.7	[5.7, 12.8]	
*≤2 pregnancies*	360	47	14.7	[10.9, 19.5]	
*>2 pregnancies*	717	57	8.7	[6.6, 11.5]	0.018
***Syphilis***					
*RPR negative*	2,192	256	12.0	[10.5, 13.7]	
*RPR positive*	229	22	8.1	[5.0, 12.8]	0.101
***HIV Infection***					
*Negative*	2,084	240	11.8	[10.3, 13.5]	
*Positive*	276	30	11.1	[7.5, 16.1]	
*Indeterminate*	61	8	10.4	[4.6, 21.8]	0.917
***Depression***					
*No*	2,060	247	12.0	[10.5, 13.7]	
*Yes*	360	30	9.4	[6.4, 13.7]	0.241
***PTSD***					
*No*	2,138	257	12.0	[10.5, 13.6]	
*Yes*	283	21	9.3	[5.9, 14.3]	0.271

### Factors associated with chronic Hepatitis B infection

Adjusting for other factors, older age (OR: 0.570; 95%CI: 0.368–0.883) and higher educational level (OR: 0.598; 95%CI: 0.412–0.868) were associated with reduced odds of chronic HBV infection (Table 3). Adjusting for other factors, being male (OR: 1.639; 95%CI: 1.007–2.669) and ever having been abducted (OR: 1.461; 95%CI: 1.055–2.023) were associated with increased odds of chronic HBV infection. In addition, among women, having 1 or 2 pregnancies (compared to none or >2) was associated with increased odds of chronic HBV infection (OR: 1.764; 95%CI: 1.009–3.084).

**Table 3:** Multivariable analysis for factors associated with chronic HBV infection (N = 2421).

*Characteristics*	*Total*	*Chronic HBV*	*Unadjusted*			*Adjusted (Final Model)*		
	N	n	Odds Ratio	95% CI	p-value	Odds Ratio	95% CI	p-value
***Overall***	2,421	278						
***Age group***								
*13–34 years*	1,945	242	Ref			Ref		
*35–49 years*	476	36	0.618	[0.407, 0.940]	0.025	0.570	[0.368, 0.883]	0.012
***Sex***								
*Female*	1,409	133	Ref			Ref		
*Male*	1,012	145	1.369	[1.028, 1.822]	0.031	1.639	[1.007, 2.669]	0.047
***Marital Status***								
*Not in union*	1,141	150	Ref					
*Currently in union*	1,280	128	0.750	[0.563, 0.999]	0.050			
***Education level***								
*No Education*	354	52	Ref			Ref		
*Now or ever at school*	2,067	226	0.716	[0.495, 1.035]	0.075	0.598	[0.412, 0.868]	0.007
***District of residence***								
*Amuru*	709	70	Ref					
*Gulu*	1,159	131	1.154	[0.820, 1.623]	0.412			
*Nwoya*	553	77	1.442	[0.997, 2.086]	0.052			
***Community type***								
*Displaced*	329	32	Ref					
*Permanent*	1,143	143	1.259	[0.818, 1.937]	0.296			
*Transient*	949	103	1.105	[0.694, 1.760]	0.674			
***Ever Abducted***								
*No*	1,833	199	Ref			Ref		
*Yes*	588	79	1.329	[0.962, 1.836]	0.084	1.461	[1.055, 2.023]	0.023
***Sexually abused in abduction***								
*No*	457	61	Ref					
*Yes*	99	10	0.71	[0.32, 1.60]	0.412			
***Sexual assault in context of war***								
*No*	2216	262	Ref					
*Yes*	205	16	0.69	[0.38;1.25]	0.224			
***Pregnancies***								
*Never been Pregnant*	332	29	Ref			Ref		
*≤2 pregnancies*	360	47	1.807	[1.038, 3.146]	0.037	1.764	[1.009, 3.084]	0.047
*>2 pregnancies*	717	57	1.003	[0.587, 1.713]	0.991	0.968	[0.548, 1.708]	0.910
*Men*	1,012	145	1.656	[1.019, 2.690]	0.042	1.000		
***Syphilis***								
*RPR negative*	2,192	256	Ref					
*RPR positive*	229	22	0.642	[0.380, 1.086]	0.098			
***HIV Infection***								
*Negative*	2,084	240	Ref					
*Positive*	276	30	0.925	[0.586, 1.460]	0.737			
*Indeterminate*	61	8	0.867	[0.362, 2.077]	0.749			
***Depression***								
*No*	2,060	247	Ref					
*Yes*	360	30	0.765	[0.490, 1.194]	0.238			
***PTSD***								
*No*	2,138	257	Ref					
*Yes*	283	21	0.750	[0.448, 1.256]	0.274			

### Focus on women of childbearing age

#### Chronic HBV prevalence and pregnancy

Among 1,353 women of childbearing age (13–45 years old), 129 (10.5%) had a chronic HBV infection (Table 4). Median age of women with chronic HBV was lower than among those without chronic HBV (24 vs. 25; p = 0.039). One-hundred (11.1%) women who had ever been pregnant had chronic HBV. In addition, 19 (15.0%) currently pregnant women had chronic HBV infection.

**Table 4:** Chronic HBV Infection among women of child-bearing age (13–45 years).

Variable		Chronic Hepatitis B					
		Negative (n = 1224; 89.5%)	Positive (n = 129; 10.5%)	Total	OR^1^	95% CI	p-value
				(n = 1353)			
**Age (IQR)**		25.0 (19.0–32.0)	24.0 (19.0–28.0)	25.0 (19.0–32.0)	0.970	[0.959, 0.991]	0.039^2^
**Ever Pregnant**	No	302	29 (8.7%)	331	Ref		
	Yes	922	100 (11.1%)	1022	1.305	[0.79,5 2.142]	0.291
**Currently Pregnant**	No	1087	110 (10.1%)	1197	Ref		
	Yes	125	19 (15.0%)	144	1.579	[0.885, 2.819]	0.119
**Ever had sex**	No	201	19 (15.1%)	220	Ref		
	Yes	1016	110 (10.8%)	1126	1.239	[0.690, 2.224]	0.472

^1^ Unadjusted odds ratio.

^2^ P-value obtained using the Wilcoxon rank-sum test.

-Sampling weights were used for estimation of proportions and ORs as well as in calculating p-values.

#### Vaccination

Among 1,353 women of childbearing age, only 141 (12.3%) had been vaccinated against HBV (Table 5). There was no significant difference in median age between women who had been vaccinated against HBV and those who were not. Women in Gulu and Nwoya had significantly higher odds of being vaccinated than women in Amuru (OR: 4.22; 95%CI: 2.46–7.23 and OR: 1.88; 95%CI: 0.99–3.58, respectively). Women living in a transient community were less than half (OR: 0.35; 95%CI: 0.18–0.68) as likely to have been vaccinated than those living in a permanent or displaced community. Among 1,022 women who had ever been pregnant, 911 (87.1%) had not been vaccinated against HBV. Among the 144 currently pregnant women, only 10 (5.8%) had been vaccinated against HBV.

**Table 5:** HBV Vaccination among women of child-bearing age (13–45 years).

**Variable**		**HBV Vaccination**					
		No	Yes	Total	OR^1^	95% CI	p-value
		(n = 1212; 87.7%)	(n = 141; 12.3%)	(n = 1353)			
**Age (IQR)**		25.0 (19.0–32.0)	25.0 (20.0–31.0)	25.0 (19.0–32.0)	1.011	[0.990, 1.034]	0.233^2^
**Ever Pregnant**	No	301	30 (10.1%)	331	Ref		
	Yes	911	111 (12.9%)	1022	1.317	[0.821, 2.114]	0.252
**Currently Pregnant**	No	1066	131 (13.2%)	1197	Ref		
	Yes	134	10 (5.8%)	144	0.403	[0.182, 0.893]	0.021
**District**	Amuru	358	22 (4.6%)	380	Ref		
	Gulu	576	94 (17.0%)	670	4.219	[2.461, 7.231]	<0.001
	Nwoya	278	25 (8.4%)	303	1.884	[0.991, 3.582]	0.054
**Community Displacement Status**	Displaced	152	22 (12.1%)	174	Ref		
	Permanent	597	90 (16.0%)	687	1.375	[0.806, 2.344]	0.242
	Transient	463	29 (4.6%)	492	0.349	[0.178, 0.684]	0.002

^1^ Unadjusted odds ratio.

^2^ P-value obtained using the Wilcoxon rank-sum test.

Note: Sampling weights were used for estimation of proportions and ORs as well as in calculating p-values.

## Discussion

### Overall findings

We observed a high prevalence of chronic HBV infection (11.5%) among conflict-affected people living in mid-Northern Uganda. Our estimates are similar to what was found during the Uganda HIV/AIDS Sero-Behavioural Survey of 2004–2005 where 10.3% of participants were HBsAg-positive [4], and in the Uganda Population-based HIV Impact Assessment Survey where people with Acholi ethnicity had the highest surface antigen prevalence at 9.2% [12]. Overall prevalence of chronic HBV infection in the current study was higher than the surface antigen prevalence found in mid-North region during UPHIA 2016–2017 survey, which was 4.6% among people aged 0–64 years [12]. Our findings indicate that a substantial proportion of people living with untreated HBV in the North, for whom treatment is urgently required.

Recent case study modelling of potential impacts of HBV intervention strategies in Uganda suggests that the biggest reductions in chronic HBV prevalence are possible with a test and treat approach addressing existing infections, similar to that used in the HIV response [23]. At present anti HBV treatment (tenofovir) is not widely available publicly in Northern Uganda and is expensive privately. Instead, physicians prescribe a combination pill Truvada (tenofovir and emtricitabine) which is meant for HIV treatment. Yet, significant barriers to care are indicated by the high median viral load observed in this study, contributing to community transmission, illness and death. Given our finding that more than 1 in 10 participants in *Cango Lyec* were living with chronic HBV, alongside other evidence that the mid-Northern region of Uganda is the most affected, we recommend establishment of Northern Uganda Liver Wellness Centres in order to reach the WHO strategic targets aimed at preventing HBV transmission, morbidity, and mortality.

### Factors associated with chronic HBV

Factors associated with reduced odds of chronic HBV infection included older age and higher educational attainment, consistent with previous studies [4, 6, 14]. Being male and having experienced abduction during the civil war were associated with increased odds of HBV infection. Elevated risk of HBV infection among men in Cango Lyec is consistent with higher prevalence among men compared to women in Uganda overall (5.4 vs 3.0%) [12] and over time [4], as well as sub-Saharan Africa more generally [9]. Increased odds of chronic HBV among those who had been abducted during the Uganda’s civil war, however, is a novel finding. This may be accounted for by higher incidence of HIV among *Cango Lyec* participants who had been abducted, compared to those who had not [21]. Previous research has demonstrated having HIV contributes to chronicity of newly acquired HBV infections [9]. In addition, evidence indicates that HIV–HBV co-infection promotes a more aggressive disease course of HBV, including increased risk of acute liver failure, progression of fibrosis and cirrhosis, and hepatocellular carcinoma [9]. While not yet well understood, linkages between war-related experiences and HBV have also recently been observed in study involving 1000 people living with HIV attending St Mary’s Hospital in Gulu, Northern Uganda [24]. Given high liver-related morbidity and mortality among individuals who are co-infected with HIV-HBV, availability of trauma-informed antiviral care for those who have survived the war–especially those who experienced abduction–is critical [5, 9].

### Prevention of mother-to-child transmission

Risk of becoming a carrier of chronic HBV is higher in the perinatal period, compared to in adulthood [6, 9]. Our observation that few (n = 3) of all infections were acute, alongside evidence of higher prevalence among younger Cango Lyec participants, suggests that the primary mode of transmission of infections observed in this study is mother-to-child [9]. Prevalence of chronic HBV was 10.5% among women of childbearing age, just slightly lower than surface antigen prevalence observed among women attending antenatal care at two hospitals in Gulu District [6]. We found that approximately 90% of women who had ever been pregnant remained unvaccinated, including all but 7% of women who were currently pregnant at the time of the study. In addition, experiencing 1 or 2 pregnancies (compared to none or >2) was associated with increased odds of chronic HBV infection. These findings affirm that prevention of mother-to-child transmission is critical to the HBV response in Northern Uganda. However, at present, HBV testing is not a routine part of antenatal care offered in the country as a whole [6]. Therefore, we add our voices to existing calls for antenatal care in Northern Uganda to include HBV screening and antiviral treatment for chronic HBV infection to support the health and wellbeing of mothers as well as prevent mother-to-child transmission [6, 9, 12]. In addition, for expectant mothers who are HBV-negative, previous studies have determined that HBV vaccination during pregnancy is safe and should be offered [9].

### Vaccination

Only a small proportion of Cango Lyec participants eligible for HBV vaccination had been vaccinated. At the time of the study (2011/12), just 10.9% of participants had been vaccinated for HBV and 45.7% remained susceptible and are therefore eligible to receive vaccination. Since 2002, the Uganda National Expanded Program on Immunizations (UNEPI) has recommended three doses of HBV vaccine, initiated at six weeks or first contact after that age [14, 25]. Yet, nearly 20 years on from the introduction of HBV childhood immunization, our findings demonstrate that the greatest proportion of Cango Lyec participants living with chronic HBV are under 35 years old. Others have raised concerns that waiting until six weeks of age limits the ability to prevent mother-to-child transmission and allows for transmission through close contacts in the first weeks of life [6, 14]. In 2015, the country further initiated a scaled-up testing and vaccination program of adolescents and adults, including a focus on districts in Northern Uganda [8]. However, recent modelling suggests that catch-up vaccination in adults has negligible effect on population prevalence, while routine neonatal vaccination and prevention of vertical transmission are likely to have marked and sustained impact [23]. This is also evidenced in a recent literature review which highlighted two studies indicating that providing newborns with HBV hyperimmune globulin as well as HBV immunization within 24 hours of delivery prevents vertical transmission in 80–95% of cases [9]. As a result, we add our voices to calls for making birth-dose HBV vaccination followed by two subsequent doses widely accessible as part of standard care particularly in mid-Northern Uganda [6, 9, 14].

### Strengths and Limitations

Of the 291 individuals who tested positive to HBsAg at baseline, only 184 provided a second sample at a follow-up round. As a result, we were unable to confirm chronicity on some samples based on a greater than 6-month follow-up sample but we were able to use Anti-HbcAb, Anti-IgM-HbcAb and Anti-HbsAb test results to make this classification. Nevertheless, misclassification of chronic infections cannot be completely ruled out. This study did not seek to assess occult HBV infection and no ALT (Liver Function Test) was undertaken. As a result, the frequencies of HBV infection overall presented in Table 1 may have been under or overestimated. Given our deliberate focus on chronic HBV infection, some participants with acute HBV infection at the time of sample collection but who may have later developed chronic infection were included in the comparison group, and this may have led to an underestimate of the association between risk factors and chronic HBV infection overall. In addition, in the cross-sectional study design the exposures and outcome were simultaneously measured and therefore it is difficult to establish temporal relationship between potential HBV related vulnerabilities and the infection. The study’s main strength is a complete set of antibody and antigen tests done to allow accurate classification of HBV infection status, as well as the large and representative sample with high response rates.

## Conclusion

Chronic HBV and other viral hepatitis are public health crises contributing to mortality and morbidity equivalent to or greater than diseases such as malaria, tuberculosis, and HIV [9]. Post-conflict dynamics in Northern Uganda may contribute to increased transmission and barriers to screening, vaccination, and treatment among people who survived the war, accounting for the high prevalence observed in this study. It is critical that those living with chronic HBV are identified and receive treatment. In particular, focus on women of childbearing age, to ensure prenatal screening and antiviral treatment during pregnancy to prevent mother-to-child transmission, is essential. Finally, our findings affirm calls for shifts to neonatal birth-dose vaccination to enhance lifetime immunity from this vaccine-preventable disease.
